# Emotion in public involvement: A conceptual review

**DOI:** 10.1111/hex.14020

**Published:** 2024-03-19

**Authors:** Kristin Liabo, Lauren Asare, Philip Ruthen, Julia Burton, Pamela Staunton, Joanne Day

**Affiliations:** ^1^ Department of Community and Health Sciences, Faculty of Health and Life Sciences, University of Exeter Medical School University of Exeter Exeter UK; ^2^ Exeter Lived Experience Group (LEG), Department of Psychology, Faculty of Health and Life Sciences University of Exeter Exeter UK; ^3^ National Institute for Health Research Applied Research Collaboration South West Peninsula Public Engagement Group (PenPEG) University of Exeter Exeter UK

**Keywords:** emotional labour, engaged research, patient and public involvement

## Abstract

**Background:**

Experiential knowledge can aid in designing research by highlighting what an idea looks like from a patient and carer perspective. Experiential knowledge can be emotional, and this can create challenges at formal research meetings.

**Objective:**

The aim of this study was to consider the role of emotions in public involvement.

**Methods:**

This is a conceptual review informed by relevant literature and reflection within the author team. A structured Scopus search was conducted in November 2021 and December 2022, identifying 18 articles that presented findings from patient and public involvement (PPI) research related to ‘emotion’. We complemented the search with theory‐generating articles related to the role of emotion and emotional labour in human life.

**Findings:**

Study findings from the structured search were tabulated to identify recurring themes; these were as follows: emotional connections to the research topic can cause stressful as well as cathartic experiences of PPI, ‘emotional work’ is part of PPI when people are contributing with their experiential knowledge and the emotional aspect of ‘lived experience’ needs to be recognised in how PPI is planned and facilitated. These points were considered in relation to theoretical works and experiences within the author team.

**Discussion:**

‘Emotion work’ is often required of public collaborators when they contribute to research. They are asked to contribute to research alongside researchers, with knowledge that often contains emotions or feelings. This can be both upsetting and cathartic, and the environment of the research study can make the experience worse or better.

**Conclusions:**

The emotional component of experiential knowledge can be challenging to those invited to share this knowledge. It is imperative that researchers, research institutions and health and care professionals adjust research meeting spaces to show an awareness of the emotional labour that is involved in PPI.

**Patient or Public Contribution:**

This review was initiated after a meeting between carers and family members of residents in care homes and researchers. The review is co‐written by a group of three researchers and three carers and family members. Regular online meetings were held during the draft stages to incorporate people's views and ideas. Data extracted from the review were presented to the group of public collaborators in a variety of formats (e.g., posters, slideshows, text and verbally) to facilitate shared sense‐making and synthesis of the literature.

## 1 INTRODUCTION

This review examines the role of emotion in public involvement. To explore emotion as an integral concept within public involvement, we draw on relevant literature and own experience from being involved in, and facilitating public involvement in, health and care research. We conclude with practical recommendations on how to make research meetings feel safe and appropriate for people who have an emotional connection to the research topic.

Different terms are used for activities that bring people with lived experience of healthcare together with researchers and service providers. Here, we use the acronym patient and public involvement (PPI) for when people with lived experience are asked to contribute to research and service planning, design, dissemination and governance. We use the term public collaborator for people who are invited to health research teams due to their lived experience of the research topic.

The inspiration for this review arose within the University of Exeter and Care Homes Knowledge (ExCHANGE) Collaboration. This project brought together staff, residents, family members and other care home stakeholders to build capacity for care home research.^1^ The Family and Friends Involvement group within the ExCHANGE project initially consisted of seven people who were supporting care home residents, as family members and friends. Our original plans for involving care home residents changed due to the pandemic. While residents were eventually involved in the project, they did not attend the online involvement group meetings. Some of the seven members had met each other and the researchers before, at involvement meetings about other research topics, while three were completely new to research involvement.

At the first Family and Friends Involvement group meeting, members met with three researchers to discuss priority areas for care home research. At the meeting, when considering research topics, people shared knowledge gained from visiting and caring for parents and spouses, and close friends. Drawing on their experiences of guilt and loss, as well as examples of good care home practices, people considered areas where research could contribute to knowledge about care homes and how to care for people living in them.

The group later reflected on the emotional nature of the conversations at this meeting and how emotion had strengthened the force of what people said. Emotional stories and viewpoints brought about a sense of importance and urgency in the researchers in a way that more neutral accounts did not. Emotional stories also bound the group together, even though they had not previously met as a group. People found common ground for their involvement in research and felt connected through sharing emotional experiences. Through our reflections, we became more aware of the role that emotion can play in public involvement. While it had not been a negative experience to share emotion at *this* meeting, we imagined that it could be experienced as problematic in a different setting.

We write as a collective, where some of us have worked in co‐production, peer research and public involvement for more than twenty years, as researcher or public collaborator. We have seen emotions surface at research meetings and involvement workshops, by public collaborators, researchers and healthcare providers. Research topics can bring about emotion, but so can differences in opinion, power imbalances and how a meeting is organised and run. What we saw in our care home meeting was emotion being shared as an integral part of experiential knowledge, and that is the focus of this conceptual review.

## 2 REVIEW METHODS

This review is conceptual and configurative because it aimed to provide rich consideration of a component of PPI rather than a conclusive answer to a specific question.^2^ The review draws on research findings and theoretical works on the concept of emotion across different disciplines, to inform PPI theory and practice. The review methods are outlined in Box 1.

Box 1 **Review methods**

**Table:** 

A structured Scopus search was conducted in November 2021, with no date limits, and updated in December 2022. Search terms: (TITLE‐ABS‐KEY (‘patient and public involvement’ OR ‘patient involvement’ OR ‘public involvement’ OR ‘public engagement’ OR ‘service user involvement’) AND ABS (emotion OR emotional)) = 355 hits in total. Titles and abstracts were screened on relevance, and full texts were scrutinised where needed. We did not specify study design, and studies were not critically appraised. Findings from 18 studies were tabulated on title, abstract and findings related to PPI being emotional or the role of emotion in PPI. The tabulation enabled us to identify recurring findings across the articles. A truncated version is presented in Table 1. In addition, we identified theory‐generating articles related to the role of emotion and emotional labour in human life from previous work and studies within the author team. LA and KL have studied sociology, PR is a published author and JD is a psychologist. We specifically sought seminal articles on emotional labour and works that have considered what emotion is and how it connects to experiential knowledge. The author team considered the themes arising from the structured literature search and the theoretical contributions from sociology, psychology and literature studies in view of their own experiences of PPI. The integration of knowledge was through deliberation at meetings, followed by the circulation of the emerging draft article, followed by another meeting, and so on, until we reached a consensus on the article's main themes and key messages for practice. Integration was aided by presentations drawing out key themes from the literature and careful note‐taking at meetings to capture the author team's interpretations of these. The chosen emphasis in this review was driven by the experiences and knowledge of the author team, combined with the themes emerging in the PPI‐specific literature, but we have not excluded any findings from the literature in our synthesis.

## 3 EXPERIENTIAL KNOWLEDGE IN RESEARCH: THE IMPORTANCE OF EMOTION

The structured literature search uncovered 18 articles (see Table 1). We next present an integrated synthesis of the findings from these studies with theoretical writings related to emotion. The overarching themes from the PPI literature are that an emotional connection to the research topic can cause stressful as well as cathartic experiences, PPI requires ‘emotional work’ and researchers should recognise the emotional aspect of ‘lived experience’. To integrate this with the theoretical works, we have chosen to focus on experiential knowledge as holding a central role in PPI. We discuss the emotional side to experiential knowledge and how emotion and formality can collide in the practical setting for PPI: the research meeting.

**Table 1:** Studies identified in the structured search.

#	Reference	Key findings on emotion in PPI
1	Allen et al.^3^	PPI contains emotional work. Researchers need to be offering safe spaces for sharing knowledge by establishing trust, careful planning and working with group dynamics. Using lived experience/experiential knowledge can be challenging. It is important to address power imbalances and that power is shared within research projects. The team assessed their own accounts against NIHR Involve frameworks for PPI/co‐production.* None of those frameworks included emotional labour, but this was a strong theme for the Partners 2 writing collaborative.
2	Carlsson et al.^4^	Acknowledges the multi‐dimensional nature of emotions and alludes to their context‐specific ability to be either stressful or liberating. It is simultaneously emotionally difficult to re‐experience a traumatic event, and yet, it also served as a chance to process experiences, causing mixed feelings about PPI in the respondents.
3	Chambers et al.^5^	PPIE could have high emotional cost for public collaborators. Coming together in groups was reassuring/confidence boosting; being with others with same condition was beneficial, e.g., through networking, making friends, gaining mutual support. Working together gave a sense of solidarity and comradery. Some public collaborators said that PPI improved their relationship with their illness, and helped them to learn more about themselves and how to live with illness. Despite challenges, involvement was ‘fun’.
4	Clay and Misak^6^	Respondents spoke about the exposing and vulnerable nature of sharing painful memories with researchers during the process of PPI. Lack of experience with public speaking caused discomfort about speaking on sensitive and highly personal topics.
5	Davies^7^	Explores the idea of PPI meetings being ‘emotion‐laden’, and how this aspect may be ‘embodied’ through the physical environment in which they take place. The choice in venue can have an influence on effectiveness of PPI – different ‘sites’ can produce different emotions. PPI meetings deal with experiences and knowledges that are both ‘lay’ and scientific.
6	Froggatt et al.^8^	Emotional components as well as practical considerations (e.g., language use) presented challenges during PPI. Accessing the views of those who may have advanced illness and considerable disability can be challenging, and involvement can be time‐consuming and resource‐intensive for both researchers and public contributors.
7	Hitchen et al.^9^	Carer participants spoke about the emotional aspect of involvement, but this was not acknowledged by professionals. Meetings were experienced differently by professionals and service users. Depersonalisation was a recurrent theme in organisational language with which service users and carers found it hard to identify. Concerns were voiced about agendas focused on organisational rather than service‐user needs. Importance was placed on the organisation rather than people.
8	Johnson et al.^10^	Relationship‐building created a safe environment for discussing sensitive topics, although public members felt that greater consideration of emotional support was needed. What helped: careful consideration of emotional support when broaching sensitive topics; the ability to work flexibly with people living in complex and unpredictable circumstances; and an emphasis on involving people across the diversity of our field who have experience relevant to the specific research project. Debrief before or after meetings provided emotional support.
9	Ludwig et al.^11^	Barriers to involvement due to diminished capacity for comprehension, heightened emotional distress due to subject matter or pathophysiology. Provision of practical and emotional support, and comfort (e.g., refreshments, quiet spaces) are facilitators to involvement. Researchers described exposure and sensitisation to the lived experience of illness and suffering, and yet failed to acknowledge the concomitant emotional labour and associated burden.
10	Ludwig et al.^12^	Emotional impact of patient‐partners learning about poor prognoses or unsuccessful treatment outcomes related to their own conditions. Responsibility of researchers to be mindful of how information is presented. Formal debriefing mechanisms are a way to respond to emotional distress, particularly when patient partners had progressive and/or palliative illnesses.
11	Mackintosh et al.^13^	While the women included in the study reported emotional burden associated with participation, this was overshadowed by the ‘cathartic experience of sharing and being heard’. ‘For those women that did participate, within the democratic tradition, involvement is seen as something that has intrinsic value in and of itself’. (p. 656)
12	Mathie et al.^14^	Acknowledges that providing emotional support for collaborators is an important part of the PPIE process. Excerpt from a public collaborator included in the ‘Emotional Support’ section—‘The actual intellectual, emotional property to that person's experience is being given freely, but there is a price every time, every time you go in and delve inside yourself to give, it is a gift, because not everyone can do it, not everyone wants to do it’. (PPI contributor) (p. 6/7)
13	Mitchell et al.^15^	Managing group dynamics could be difficult, different working/comprehension speeds leading to frustration within the group. Building the trust and sharing difficult information, as well as managing emotions for researchers and PPI members can be emotionally challenging.
14	Montgomery and Donnelly^16^	Support needed for projects involving service users, e.g., administrative and emotional support. Researchers should be trained to take account of the difficulties when dealing with crises during PPI.
15	Sitzia et al.^17^	Feelings held by collaborators, the nurse specialist and a cancer information officer felt emotional when service users talked about their personal experiences during meetings. Public collaborators can feel an emotional attachment to a PPI group. PPI can provide emotional support.
16	Skovlund et al.^18^	PPI requires ‘tacit work’. ‘PPI is a time consuming, emotional, cognitive, and practical effort’ (p. 11) and ‘PPI in health research requires huge investments (e.g., time, financial resources, and emotional and intellectual effort), not only by research organisations and teams, but also by the patients who get involved’. (p. 13)
17	Todd et al.^19^	Emotional labour and possible burden for researchers facilitating PPI. ‘PPI facilitators are key in all PPI processes, from being a gatekeeper for patients and the public, to facilitating stakeholders’ conversations, to making recommendations for service improvements, it appears important to provide them with adequate instrumental and emotional support’. (p. 469)
18	Wright et al.^20^	Emotional demands of PPI members being involved in research can raise ethical issues. Appropriate emotional support is necessary, e.g., supervision. Emotional demands, e.g., ‘raising issues with participants receiving palliative care can be challenging due to a fear of asking inappropriate or potentially disturbing questions’. (p. 824)

Abbreviations: PPI, patient and public involvement; PPIE, patient and public involvement and engagement.

* Co‐production is a term used for when research is collaboratively created between different stakeholders and researchers.

### 3.1 Experiential knowledge

Knowledge gained from using services, living with a condition and receiving care is increasingly considered important to the planning of health and care research.^21^, ^22^, ^23^ This type of knowledge is often called ‘experiential knowledge’, defined as ‘truth learned from personal experience.^24^ ^,p.446^ This is not simply fragments of feelings that we all experience as human beings, but insight and wisdom gained through reflecting on certain experiences in depth.^24^, ^25^ Experiential knowledge can be emotional and have an emotional source.^26^ Experiences such as grief or relief after treatment can prompt actions and reflection that lead someone to develop experiential knowledge.

‘Experiential knowledge’ is sometimes contrasted with professional knowledge, which is developed within the context of formal education. While the value of experience is rarely contested, there is a knowledge hierarchy within research where personal views and opinions sit on the lowest rungs. However, ‘experiential knowledge’ is not brought in to replace scientific knowledge. It is brought in to complement it, with a recognition that there are many types of knowledge needed in health and care research.^27^

For example, scientific knowledge is used to develop medicines that reduce pain or treat conditions in older people. This is fundamentally different to knowledge gained from managing medicines in care homes, or administering them to a family member or knowledge about which innovations are most needed to improve the quality of life in care homes. Experiential knowledge can help design accessible and inclusive research because it can highlight what an idea looks like from the perspective of patients and carers. Experiential knowledge can also help plan the research so that it is more attractive to potential participants, thereby improving research recruitment rates and, in turn, research validity.^28^

### 3.2 Emotion in experiential knowledge and research

Emotions are part of what it means to be human, something that we sense in our bodies when we see an image, think about something or share a memory. Hochschild^29^ uses the terms emotion and feeling interchangeably; ‘although the term “emotion” denotes a state of being overcome that “feeling” does not’.^29^ ^,p.551^ Others have distinguished between ‘affect’ and ‘emotion’: affect is evoked by lived experiences and often remains unspoken but gives rise to emotions. ‘Although deeply rooted in affective, social experiences from the past, emotions are evoked in the present, are more subjective and mediate people's behaviour and agency’.^26^ ^,p.1140^

There is a plethora of works across disciplines that investigate and theorise the role played by emotion in social interactions, human systems, religion, psychology, health and the arts.^30^, ^31^, ^32^, ^33^ Particularly relevant to public involvement are works that consider emotional knowledge, and emotion's role in knowledge production. For example, in literary scholarship, Eliot suggests that literary composition is a ‘stimulated state of mind when an intense, purposive intellect brings feelings or emotions into new order.’^32^ The triggered passing feeling, or enduring emotional state arising directly from incidents or settings in a person's personal life, transforms into something impersonal and objective, yet emotional, when transferred onto the published page: ‘Poetry is not a medium to unleash raw emotion in an artless, uncontrolled and undisciplined way’.^32^ In other words, literature can be truthful, and while it is often emotional, it is also objective because it evokes memory of experiences that have universal application beyond the individual author. This connection between emotion and truth was seen in the PPI literature too, with emotion considered core to contributions by people with lived experience.^9^

The term ‘lay’ knowledge from sociology also touches on the truth that can be derived from experience, as the articulation of meanings people ascribe to health, illness, disability and risk.^34^, ^35^ This brings us back to the starting point of this review: experiential knowledge as insight and wisdom gained through reflection.^25^ Experiential knowledge can contain emotion as a core component, and sharing experiential knowledge can also be emotional due to the intertwining of emotion within that experience and within that knowledge.

In the management literature, ‘emotional knowledge’ is described as knowledge gained through inter‐personal connections, which is in turn applied to create a ‘good’ workplace. A manager's personal and organisational experiences intertwine in their managerial work, where they focus both on the technical needs of the organisation and the psychological needs of the individual workers.^36^, ^37^ Another relevant concept is emotional labour, or emotion work. This is not simply the act of suppressing emotion, but includes expression of, and managing, emotion in response to a situation.^37^, ^38^

The PPI research literature supports the notion that providing experiential knowledge to research can be an emotional experience.^5^, ^9^, ^10^, ^13^, ^14^, ^15^, ^16^, ^17^, ^18^, ^20^ Sometimes, this means a painful emotional experience: ‘…you might cope with it in a meeting or that setting, but then it sets off a whole train of thought and sort of sad reflections when you leave that meeting, and um, the impact can stay with you.’^10^ ^,p.156^ Involvement in research can be triggering, but when it is initiated within a supportive environment, the positive can outweigh the negative.^5^, ^13^, ^15^ Interestingly, some of the positive experiences from public involvement also include emotions: relief and liberation through sharing experiences,^4^ feelings attached to a PPI group^17^, ^18^ and supportive feelings through peers.^11^

### 3.3 Emotion in research meetings

Public involvement primarily happens in meetings between people: researchers with formal knowledge of how to conduct studies on particular topics meet people with lived experience of the same topic. Sometimes, these meetings are informal and include just one researcher and a public collaborator, perhaps at an early stage, to bounce research ideas. Other times, they are very formal, for example, a study steering committee with a chair, an agenda, time slots for each agenda item and recorded minutes, with a focus on governance or study management. Public collaborators can be in the minority at committee‐styled meetings and in the majority at meetings that focus on their specific contributions from lived experience, for example, in a PPI workshop. People attending are likely to prepare differently for meetings depending on whether public collaborators or researchers are in the majority, where the meeting is held and what the meeting aims to achieve.

In a formal research meeting, it is likely that public collaborators will find it necessary to manage their emotions, because the official and unofficial meeting ‘rules’ invite dispassionate contributions over passionate ones. Social situations affect both what people feel, and what people think and do about what they feel.^29^ ^,p.552^ People are likely to interpret a round‐table discussion in an office building, with people in suits, a named meeting chair and a timed agenda, as an inappropriate place to cry or laugh, and the meeting context is likely to invoke ‘feeling rules’.^29^ These rules are tacit understandings of what is appropriate behaviour and what one should expect to experience, rather than written rules of conduct. Feeling rules within formal meetings mean that those who are experiencing strong emotions are likely to induce or inhibit their feelings to make them appropriate to that social situation. This is what is called ‘emotional labour’.^39^ However, if emotion is an intrinsic part of experiential knowledge, excluding it can potentially reduce the impact of PPI because only part of the story is shared, and it can leave people who are offering lived experience feeling overwhelmed or excluded, leading people to withdraw.^5^

There are further contextual issues aside from ‘feeling rules’ that can make meetings emotional for people with lived experience. A review by Chambers et al.^5^ ^,p.997^ concluded that ‘involvement itself also caused distress’. If the meeting is poorly facilitated, if health conditions and/or experience of services are discussed insensitively or if there are unrealistic expectations of people's available time, people can feel emotionally exhausted from involvement.^3^, ^5^, ^11^, ^12^ For people with lived experience, these demands can be intrinsically linked to why they are at the meeting in the first place: their health condition.

A variety of factors impact on how experiential knowledge is welcomed and accommodated at a meeting. Sharing experiential knowledge and emotion might be difficult in large formal meetings, while smaller informal meetings might feel more accepting. There can also be a difference between online versus in‐person meetings, what information people receive in advance and what is asked of them. Being willing to share emotion is not typically described as being required of the public collaborator role. Although emotion can be a core part of someone's experiential knowledge, they might not wish to share emotion as part of this knowledge.

A recollection from the Family and Friends involvement group speaks to the uncertain space that can arise from emotional contributions within factual spaces: ‘I vividly remember in a [city]‐wide partnership meeting when offering a different argument on a controversial mental health agenda item and getting a stunned silence around the room because I'd delivered the points in an overly “passionate” way and from probably what I thought was an appropriate angle but was likely perceived as a bit “odd.” I mentioned this to a Community Worker who had been present afterwards, as I was still feeling awkward about it. He said, “don't worry about it at all, they expect service users to say and do odd things.” To this day I don't know if that was reassuring, or compounded the feelings of “difference”….’

## 4 DISCUSSION

The starting point for this conceptual review was our collective experience of a meeting where we found that the emotions shared as part of experiential knowledge brought people together and heightened the impact of what they said. At that meeting, the emotional experience was accepted, welcomed and acknowledged, and connected our newly formed group while strengthening our shared purpose: to identify priorities for research in care homes.

Drawing on PPI‐specific research and theoretical works on ‘emotion’, we argue that emotional knowledge and emotion work is often required of public collaborators: They are asked to contribute to the technical activity that is research, in a process designed for researchers, with knowledge that often contains emotions or feelings'.^5^, ^12^, ^38^, ^40^ The researcher might appear as the passionless deliberator and the public collaborator as the passionate enthusiast, due to the different types of knowledge that they are there to share.^7^ While a topic for discussion can be dispassionately shared through technical meeting papers, a person with lived experience of the topic might react to particular words or situations described exactly because this connects to their lived experience.^12^ Everyone at the meeting might have to exercise some emotional work, but public collaborators are likely to do more of it due to the knowledge that they are there to provide.

Sharing experiential knowledge at research meetings can be traumatising, upsetting and it can make people uncomfortable and decline further involvement.^5^, ^6^, ^9^, ^11^ Sharing experiential knowledge can also be cathartic and give people a sense of higher purpose, as they use their own experiences to improve research and, in turn, services.^4^, ^5^, ^13^, ^15^ However, it can also leave people feeling used and exploited, especially if it is a one–off meeting with no follow‐up, feedback or continuation of discussions afterwards. The contradiction between formal research spaces and emotional, experiential knowledge can foster a feeling of alienation.^38^

We conclude that there is a need to focus more clearly on emotion in public involvement planning in research work. This requires a nuanced approach.^3^ Inclusivity and accessibility are core principles for ‘good’ public involvement and this needs to guide how we approach emotional contributions. Some public collaborators will prefer to bracket emotion out and it should be their prerogative to do so. We also need to ensure that meetings with public collaborators present allow for emotion to be shared without the risk of social judgement.

Everyone in research meetings are humans, and emotions are part of the human experience, but research meeting formats are typically organised for dispassionate debate and decision‐making. Some meeting attendees might not expect emotional contributions, and some might feel that this is part of what makes formal meetings safe for them: The meeting agenda is known in advance, people can prepare their contributions accordingly and the focus of research meetings is on technical decision‐making. Sharing emotional experiences can render these meetings ‘unsafe’ for some because they expect this space to be factual. This can be true for patient and carer collaborators as well as researchers.

For some attendees with lived experience, formal meetings can prevent them from sharing their knowledge for fear of becoming emotional and thereby breaking the feeling rules at meetings. They might adapt their contributions to avoid this, but some emotional memories might be so strong that they chose not to share them at all. We propose that PPI cannot be void of emotion. Many patients become interested in research due to their negative experiences in the health and care system.^41^ This should be acknowledged and valued with systems in place to ensure support to individuals as well as sensitively run meetings'.^38^, ^42^ Trauma‐informed practices might have much to offer in this regard.^8^, ^43^ There is a call for more diversity in PPI. However, if we want more diversity, researchers must make room for people's emotional stories. At a minimum, we need to ensure that breaking the emotional rules is not dangerous or harmful to patient collaborators. The evidence base for ‘good’ involvement is growing.^7^, ^9^, ^12^, ^15^, ^38^, ^40^, ^42^, ^44^ The following practical steps can help prepare people with lived experience, and researchers, for the emotional side of PPI (Box 2):

Box 2 **Practical recommendations**

- Role descriptions for public collaborators to be agreed in advance of meetings and shared with all other attendees before the meeting, to help prepare everyone for the type of knowledge that public collaborators are asked to bring. Some public collaborators might want to highlight the emotional nature of their experiential knowledge, and others might not. The role description should be co‐written and specific to the meeting or project in question.
- Currently, research meetings are typically organised in the same way irrespective of whether there will be public collaborators there. We propose that this should change. Public collaborators need to be welcomed and cared for because they arrive with a particular type of knowledge, which is emotional in character. The meeting format needs to allow for this by acknowledging the different roles and contributions that people are coming with and the personal connection some have with the topic. Small initiatives can help, such as acknowledging lived experience in the room and why this is important, asking everyone to reduce their use of jargon, providing explanations for key terms and warm‐up exercises that enable people to build relationships.
- The meeting chair must familiarise themselves with the different kinds of knowledge and roles present at the meeting. The chair needs to acknowledge that some contributions might be emotional and ensure that this is accepted by attendees. The chair can help reassure attendees by including statements at the beginning of the meeting that acknowledge the sensitivity of the research topic. Meetings that acknowledge the human in us all are more pleasant environments, help solidify team cohesion and can facilitate decision‐making that is more grounded in everyone's experiences.
- Meeting minute‐takers should be asked to pay attention to contributions from public collaborators and record these in the minutes. The minutes can also acknowledge the different types of knowledge that were present in the meeting.
- Examples of actions that can help foster a more inclusive environment are pre‐meetings with public collaborators where the agenda and technical terms are explained, introductory rounds at the start of the meeting where everyone gets a chance to say who they are and what they can contribute with, agenda items where public collaborators are invited to share knowledge gained from their lived experience and time allocated to develop a shared language within the research team that is inclusive. This does not necessarily remove jargon, since some technical terms can provide more specificity and reduce the risk of misunderstanding, while others are useful shortcuts and enable more efficient communication.
- Capacity‐building and facilitated reflective sessions for public collaborators can help them find ways to share experiential knowledge in ways that they are comfortable with.
- Researchers, when designing research, should build in time for shared reflection and time to readjust approaches to involvement during the study. The external and internal pressures that people with lived experience may feel mean that researchers need to be reflexively aware, and need to consider how trauma‐informed involvement practices might be developed for research projects with particularly sensitive topics.^8^ Ultimately, past imbalances in power relationships can be repeated if care is not taken.^38^

## 5 CONCLUSION

We acknowledge that the emotional side of experiential knowledge can be challenging: being more inclusive and less formal requires not just a highly skilled facilitator, but efforts by everyone at the meeting to make it a welcoming space. There is also a balance between welcoming emotion when this is voluntarily shared and encouraging people specifically to be emotional. We suggest that the latter would be ethically dubious, while the former would be ethically sound. Our review suggests that emotion is an integral part of experiential knowledge, but this does not mean that all experiential knowledge must be emotional. For some public collaborators, it is detrimental to hide the emotional side of their knowledge, and for others, it is detrimental if they feel obliged to be emotional when they perhaps are not, or do not want to be.

Our review highlights the need for an ethical framework for public involvement in research. Research ethics is not required for public involvement, and this is an important principle that must be retained. Public involvement does not include data collection from public collaborators and it is important to maintain the autonomy of public collaborators and ensure that they are treated as equal partners. However, involvement standards and guidelines can, and perhaps should, address safeguarding principles and how to ensure ethical approaches to including people with lived experience as partners in research. It is all too easy to take a protectionist approach and through this exclude important voices from research.^45^ What is needed is more awareness of emotion in experiential knowledge and focus on how to support people to be public collaborators in a way that works for them.

PPI is increasingly expected within research. This means bringing experiential knowledge into technocratic research spaces. Experiential knowledge can be emotional. It is incumbent upon researchers, research institutions and health and care professionals to adjust research meeting spaces so that they recognise the emotional labour that is involved in PPI and consider adaptations to meeting environments that allow for emotion to be shared, and emotional support to be provided.

## AUTHOR CONTRIBUTIONS

**Kristin Liabo**: Conceptualisation; investigation; writing—original draft; methodology; writing—review and editing; project administration; data curation; supervision. **Lauren Asare**: investigation; writing—review and editing; project administration; formal analysis; writing—original draft. **Philip Ruthen**: Conceptualisation; writing—original draft; writing—review and editing; investigation. **Julia Burton**: Conceptualisation; investigation. **Pamela Staunton**: Conceptualisation; investigation. **Joanne Day**: Conceptualisation; investigation; writing—original draft; writing—review and editing; methodology; project administration.

## CONFLICT OF INTEREST STATEMENT

The authors declare no conflict of interest.

## Data Availability

The literature database created for this review is available from the authors.
